# Tacrolimus Pharmacokinetic and Pharmacogenomic Differences between Adults and Pediatric Solid Organ Transplant Recipients

**DOI:** 10.3390/pharmaceutics2030291

**Published:** 2010-09-09

**Authors:** Kwaku Marfo, Jerry Altshuler, Amy Lu

**Affiliations:** 1 Montefiore-Einstein Transplant Center,111 East 210th Street, Bronx, NY 10467, USA; 2 School of Pharmacy, State University at Buffalo, 110 Cooke Hall, Buffalo, NY 14215, USA

**Keywords:** tacrolimus, FK-506, pharmacokinetics, pharmacogenomics, pediatric transplant

## Abstract

Tacrolimus is a calcineurin inhibitor immunosuppressant that has seen considerable use in both adult and pediatric solid organ transplant recipients. Though there is much pharmacokinetic data available for tacrolimus in the adult population, the literature available for children is limited. Furthermore, very little is known about the pharmacogenomic differences in the two patient groups. Based on what information is currently available, clinically significant differences may exist between the two populations in terms of absorption, distribution, metabolism and elimination. In addition, inherent physiological differences exist in the young child including: less effective plasma binding proteins, altered expression of intestinal P-glycoprotein, and increased expression of phase 1 metabolizing enzymes, therefore one would expect to see clinically significant differences when administering tacrolimus to a child. This paper examines available literature in an attempt to summarize the potential pharmacokinetic and pharmacogenomic variability that exists between the two populations.

## 1. Introduction

Tacrolimus (Prograf, FK506) is a macrolide immunosuppressant first isolated from *Streptomyces* *tsukubaensis* in 1984 [1]. Since that time, tacrolimus has surpassed cyclosporine use [2] owing to reports of reduced acute rejection [3,4,5] and better tolerability. Tacrolimus exhibits its immunosuppressive effects through binding to immunophilins known as FK-binding proteins (FKBP), this complex interferes with the activity of a critical phosphatase also known as calcineurin. Under normal circumstances, calcineurin cleaves a phosphate group off NFAT (nuclear factor of activated T cells); this transcription factor then enters the nucleus and initiates the production of interleukin-2 as well as a host of other chemokines which eventually leads to T-cell replication. With the tacrolimus-FKBP complex present, dephosphorylation does not occur and T-cell proliferation is halted [1,6,7,8,9]. Additionally, tacrolimus may also inhibit B-cell production, although to a lesser extent [7]. 

Adverse events associated with both calcineurin inhibitors are vast but some dichotomy exists between the two drugs [10]. Adverse events worth noting include nephrotoxicity, hypertension, diabetes, neurotoxicity, hyperkalemia, hypomagnesemia, hirsutism and gingival hypertrophy/hyperplasia [10]. Tacrolimus seems to be associated with more neurotoxicity and diabetes but less so with cosmetic effects, such as hirsutism and gingival hyperplasia [10] when compared to cyclosporine. This is a major consideration for the pediatric population, who may be especially compromised by cosmetic side effects. Most of the adverse events associated with tacrolimus are dose- dependent thus minimizing exposure can reduce these unwanted effects, however insufficient exposure will result in acute rejection [10]. Balancing the exposure becomes a critical aspect in patient management with this narrow therapeutic index agent. Routine measurements of drug concentration are taken just preceding the next dose to obtain the trough concentration which depending on time after transplant is usually kept between 5 and 20 ng/mL [11]. 

Despite overall lack of data on immunosuppressant use in children, management of the pediatric populations remains based on the information extrapolated from the adult patient. The pediatric population has many physiological differences that can significantly alter pharmacokinetic/pharmacogenomic disposition in terms of absorption, distribution, metabolism and excretion [12]. In general the pharmacokinetics of tacrolimus display significant interpatient and intrapatient variability and are also dependent on the type of organ transplanted [13,14].

Although the stomach of a growing fetus is cellularly similar to that of the adult within 20 weeks of gestation, it appears to lag behind in terms of function for several years [15]. Intestinal motility, which plays a role in the absorption of many drugs increases with age and usually becomes similar to that of an adult by 36 weeks of age [16]. Additionally, infantile diarrhea may also affect drug disposition. Distribution in the pediatric population is often very different from the adult population because of the differences in body compartment composition. Total body water is greater in the neonate than the infant and is even less in the adult. The percentage of body weight that is represented by fat also increases with age up to one year before decreasing in the adult [17] and is an important factor when considering the distribution of lipophillic drugs. Alterations in protein binding may play a role in neonatal patients; albumin and alpha-1-acid glycoprotein levels and their affinity for ligands on binding sites are not equivalent to the adult until about one year of life [18]. Additionally presence of fetal proteins can further complicate drug binding [19].

P-glycoprotein (PGP) is an efflux pump that is expressed on the cell membrane of many tissues. It is widely known that tacrolimus is a substrate of PGP, thus age dependent variations in this proteins expression may lead to important pharmacokinetic/pharmacogenomic variability. Drug metabolism may be the most important factor when evaluating pharmacokinetic differences in the adult and pediatric patient. Phase 1 metabolism involves oxidation, reduction and hydrolysis. Phase 1 enzymes can activate or inactivate a drug and can occur before or after phase 2 metabolism, which is generally responsible for making molecules more polar and easier to excrete. In the case of tacrolmius, the drug is inactivated by cytochrome P450 enzymes located primarily in the liver and to some extent the GI tract. Metabolism of tacrolimus is primarily conducted by the 3A family of P450 enzymes, which have variable expression depending on age [20,21,22]. Additionally, many agents commonly prescribed in the transplant setting that may induce or inhibit CYP3A4 activity can have altered or unexpected effects on the exposure to tacrolimus in the pediatric patient where phase 1 enzymes are expressed at different levels from that of adults. 

Extensive clinical data suggests that pediatric patients may require higher doses of tacrolimus to obtain the same therapeutic concentrations as adults due to certain pharmacokinetic and pharmacogenomic differences. The objective of this paper is to review the four basic pharmacokinetic principles of absorption, distribution, metabolism and excretion (ADME) in relation to pharmacogenomics to examine where variability lie in the adult versus pediatric organ transplant recipient receiving tacrolimus therapy. 

## 2. Absorption

The absorption of tacrolimus occurs in the small intestine and is erratic and is decreased substantially by the presence of food. The bioavailability ranges from 5-93% with a mean of 25% in the adult population [7]. In the pediatric population Cmax occurs at 1-2 h [23,24], which is similar to what is seen in adults [11]. The mean bioavailability is reported as 31% [25] by the manufacturer. However, this data comes from a very small sample of children with ages ranging up to 13 years old. Contrarily, data from some studies suggest that expression of intestinal CYP3A may be increased in the very young pediatric population [25,26] which may lead to some differences in oral bioavailability. The interpatient pharmacokinetic variability often seen in children is multi-factorial. Differences in intestinal P-glycoprotein (PGP) and intestinal motility can explain some of the inconsistency. PGP is an energy-dependent transmembrane efflux pump encoded by the multidrug resistance 1 gene (MDR1 or ABCB1) gene. PGP has been shown to efflux up to 50% of drug back into the gastrointestinal tract [27]. Specific to tacrolimus, several reports have described an association between tacrolimus dose-adjusted trough concentrations and MDR1 genotypes, whereas others found no association. The potential role of other transporter proteins such as multi-drug resistance protein 2 remains unclear. *In vivo*, in adult kidney transplant recipients, no association was found between MDR2 polymophisms and tacrolimus trough levels. However, case reports suggest that diarrhea; especially in young patients can greatly increase oral bioavailability by decreasing exposure to PGP [28,29,30]. Unfortunately, there is not a lot of data regarding intestinal P-glycoprotein expression in the pediatric population, from the information we currently have from animal data, it seems that PGP representation is likely similar to that of an adult shortly after birth [31]. Despite several possible mechanisms that can lead to pharmacokinetic differences in absorption between adults and children, these differences are difficult to establish owing in large part to the intrinsically unreliable absorption characteristics of tacrolimus. Thus, if a real difference is present, it is likely not large enough to be clinically significant and therapeutic drug monitoring ensures that drug levels are kept in a desired therapeutic window.

## 3. Distribution

Tacrolimus is bound mainly to alpha1-acid-glycoprotein and to a lesser extent albumin as well as several other minor binding proteins [32]. Approximately 99% of the drug is bound [32] in plasma and concentration dependent distribution to erythrocytes is extensive which results in blood to plasma ratios ranging from 15:1 to 35:1 [33]. Therefore, whole blood is used to obtain tacrolimus concentrations for therapeutic drug monitoring. High levels of tacrolimus binding proteins in erythrocytes drive distribution. Aside from tacrolimus concentration, red blood cell distribution is dependent on several factors such as hematocrit, temperature and protein concentration [7,33]. Additionally, animal models suggest that tacrolimus distributes to lungs, spleen, liver, kidney, brain and muscle [34]. A study comparing pharmacokinetic differences in the adult and pediatric population noted that pediatric recipients had a volume of distribution of 2.5 L/kg nearly twice that of adults [35]. The low apparent volume of distribution is from the perspective of whole blood as it would be significantly greater based on plasma. Total clearance from the blood was also twice as large as the adult value [35]. Interestingly, a pediatric population kinetics study conducted by Zhao *et al*. in 2009 noticed that along with CYP3A activity and body weight, hematocrit was the biggest factor influencing clearance of tacrolimus [36]. Specifically a hematocrit less that 33% was associated with a significantly higher rate of clearance [36], possibly due to the greater exposure of undistributed drug to metabolizing enzymes. In pediatric patients, the reduced drug binding affinity of plasma proteins increase the fraction of unbound drug; this may be responsible for increased distribution and elimination. It is likely that the need for higher doses of tacrolimus needed to achieve the same concentration range required with more elapsed time post transplant are due to the recovery of hematocrit seen with new allograft [37]. 

## 4. Metabolism/Elimination

Elimination of tacrolimus is accomplished by metabolizing enzymes of the cytochrome P450 system. The portion of unchanged drug excreted is insignificant [33]. There are several metabolites of tacrolimus. Although some have shown *in vitro* activity; however their *in vivo* activity is still to be determined [39], and they do cross react with anti-tacrolimus antibodies that are used for immunoassays. Most of the biotransformation is performed by the 3A family of CYP enzymes with the possible minor involvement of other phase 1 enzymes [40]. The CYP3A5*1 allele is the wild type gene that displays normal enzymatic activity [41]. The CYP3A5*3 allele codes for a nonfunctional protein resulting in poor drug metabolizing in the homozygous *3/*3 patient (*i.e.*, poor metabolizers) [41]. Accordingly the *1/*3 heterozygous population are intermediate metabolizers of CYP3A5 substrates of tacrolimus [41]. The homozygous poor metabolizer genotype is reported to have a frequency of 0.83 in the Caucasian population with the single gene occurring in 0.92 of the population [36]. It has been shown that *1/*1 genotypes require twice the dose of tacrolimus to maintain desirable trough levels [42]. 

Tacrolimus is excreted primarily via the biliary route [43] and systemic clearance can range from .6-5.4 L/h/kg [44] though the range greatly depends on the type of transplant the patient underwent as well as several other variables already mentioned. Hepatic CYP3A metabolism may be the source for the greatest pharmacokinetic variation seen between adults and children. As described, CYP3A5 and to a slightly lesser extent, CYP3A4 are the primary oxidative enzymes responsible for tacrolimus elimination [45]. It has been suggested for some time that hepatic enzymatic activity is age dependent and may not be as profound in the pediatric population [46,47]. Additionally, there may be enhanced hepatic blood flow in the pediatric population in proportion to size [48]. May *et al*. found that *in vivo* 3A4 activity is highest in the younger population [47] after the 3A family comes to full maturation at about one year of age. Another study detected expression of CYP3A4 mRNA at 120% of the adult population in children older than 12 months [49]. This would explain why several studies report patients younger than six years old needing a substantially larger dose of tacrolimus to achieve similar levels [50,51]. Consequently, certain trials have reported a blood clearance of 0.14 L/h/kg in the pediatric population compared to 0.06 in adults [35].

## 5. Conclusion

Though there is scarcity of pharmacokinetic/pharmacogenomic data available for pediatric patients in comparison to the amount of adult studies; there is sufficient data to suspect that certain differences are nonetheless present. For some time, differences in hepatic enzyme activity were implicated in the apparent variability in tacrolimus clearance. It seems that these differences may be of importance especially in children under six years of age, when CYP enzyme expression may be most prevalent. In accordance to increased hepatic metabolism, intestinal metabolic clearance may be elevated for the same reason. P-glycoprotein activity may contribute to intestinal biotransformation to decreased oral absorption in pediatric transplant patients. Additionally because of the nature of immature plasma binding proteins, pediatric patients have a real difference in volume of distribution due to the greater abundance of free drug in the blood. Though much of the data agrees with these particular findings, the problem with most pediatric pharmacokinetic studies is the inclusion of a wide range of ages, anywhere from 1-18 years of age. To obtain a better understanding of kinetic/genomic principles in immunosuppressive pharmacology, larger studies with defined narrow ranges of age should be conducted and compared to results seen in adults. Fortunately, with the implementation of therapeutic drug monitoring, the effects of significant differences in pharmacokinetics/pharmacogenomics can be carefully observed and necessary adjustments to therapy can be implemented. 
